# Comparison of the safety of remimazolam and propofol during general anesthesia in elderly patients: systematic review and meta-analysis

**DOI:** 10.3389/fmed.2025.1409495

**Published:** 2025-02-07

**Authors:** Xianchun Liu, Longyi Zhang, Li Zhao, Xuelei Zhou, Wei Mao, Linlin Chen, Hongyu Zhu, Ying Xie, Linji Li

**Affiliations:** Department of Anesthesiology, Nanchong Central Hospital, The Second Clinical Medical College, North Sichuan Medical College, Nanchong, China

**Keywords:** remimazolam, elderly patients, general anesthesia, meta-analysis, propofol

## Abstract

**Background:**

Remimazolam is a novel sedative drug approved for procedural sedation and general anesthesia. Clinical trials have already explored its use in elderly patients for general anesthesia. For elderly patients with declining physical and physiological function, anesthesia safety is crucial. Most current clinical studies compare the safety of remimazolam and propofol, though the results are inconsistent. Therefore, we conducted a meta-analysis to compare the safety of remimazolam and propofol in general anesthesia for elderly patients.

**Methods:**

We systematically searched the PubMed, Cochrane Library, Embase, and Web of Science databases for all published randomized controlled trials comparing remimazolam and propofol for general anesthesia in elderly patients. We synthesized data from eligible studies using relative risk or mean difference, and analyzed differences in hemodynamic stability and adverse effects between the two drugs. Data extraction and quality assessment were performed independently by two researchers.

**Results:**

Eight randomized controlled trials involving 571 participants were included. Compared to propofol, remimazolam was associated with a lower incidence of hypotension (RR = 0.51, 95% CI: [0.33, 0.81], *I*^2^ = 18%, *p* = 0.3 > 0.1) and bradycardia (RR = 0.56, 95% CI: [0.31, 1.02], *Z* = 1.88, *p* = 0.06 < 0.05). The mean arterial pressure after induction was higher in the remimazolam group (WMD = 3.95, 95% CI: [3.197, 9.498], *Z* = 3.95, *p* < 0.00001). The remimazolam group had a higher heart rate (HR) after induction compared to the propofol group (WMD = 7.89, 95% CI: [−2.39, 18.17], *Z* = 1.5, *p* = 0.13 > 0.05), but this result was not statistically significant. Among other secondary outcomes, the remimazolam group had lower incidences of injection site pain, nausea and vomiting, and hypoxemia compared to the propofol group, and also had a shorter extubation time.

**Conclusion:**

In this meta-analysis, compared to propofol, remimazolam reduced the incidence of hypotension, bradycardia, and injection site pain during general anesthesia in elderly patients. The mean arterial pressure (MAP) and heart rate (HR) were more stable after induction. Remimazolam may be a safer sedative for elderly patients.

**Systematic review registration:**

https://www.crd.york.ac.uk/prospero/display_record.php?ID=CRD42024516950, CRD42024516950.

## 1 Introduction

With the aging population, an increasing number of elderly patients require surgical treatment (1). Elderly patients typically have reduced vascular elasticity, decreased cardiovascular reserve, and weakened compensatory mechanisms, making them more prone to hemodynamic fluctuations during anesthesia induction, which may lead to severe complications. Therefore, minimizing hemodynamic fluctuations during anesthesia and ensuring the safety of elderly patients is critical (2, 3). Anesthetic drugs are the direct factors influencing anesthesia outcomes, and different anesthetics have varying effects on patients. Propofol is a widely used sedative with advantages such as a short half-life and rapid recovery. It is an NMDA receptor antagonist, directly activates GABA-A receptors, and modulates calcium influx through slow calcium channels (4). Compared to alternative sedatives, patients using propofol typically experience faster postoperative recovery. However, the use of propofol is associated with some adverse effects, including respiratory depression, hypotension, and injection site pain (5). Using standard doses of propofol in elderly patients carries a risk of complications such as hypotension, bradycardia, and arrhythmias (6). Remimazolam is an ultra-short-acting benzodiazepine, rapidly metabolized to inactive metabolites by tissue esterases. It induces sedation quickly, offers good control over anesthetic depth, and has favorable safety, especially in terms of hemodynamic stability (7). Recent clinical studies suggest that remimazolam is comparable to propofol in terms of efficacy and safety during general anesthesia. However, other studies indicate that remimazolam has a lower incidence of intraoperative hypotension compared to propofol (8, 9). Although these studies suggest that remimazolam may be a better choice for anesthesia sedation compared to propofol, there is still a critical gap in the literature regarding the assessment of the safety and potential adverse effects of these sedatives, especially in elderly patients undergoing tracheal intubation and general anesthesia. To strengthen the existing literature and address contemporary controversies, this study conducted a comprehensive systematic review and meta-analysis to compare the safety of remimazolam and propofol in general anesthesia for elderly patients.

## 2 Methods

The meta-analysis was conducted in accordance with the Preferred Reporting Items for Systematic Reviews and Meta-Analyses (PRISMA 2020) guidelines (10). As this study is a systematic review and meta-analysis, ethical approval was not required. The study was registered in PROSPERO with the registration number CRD42024516950.

### 2.1 Search strategy

**Figure 1:**
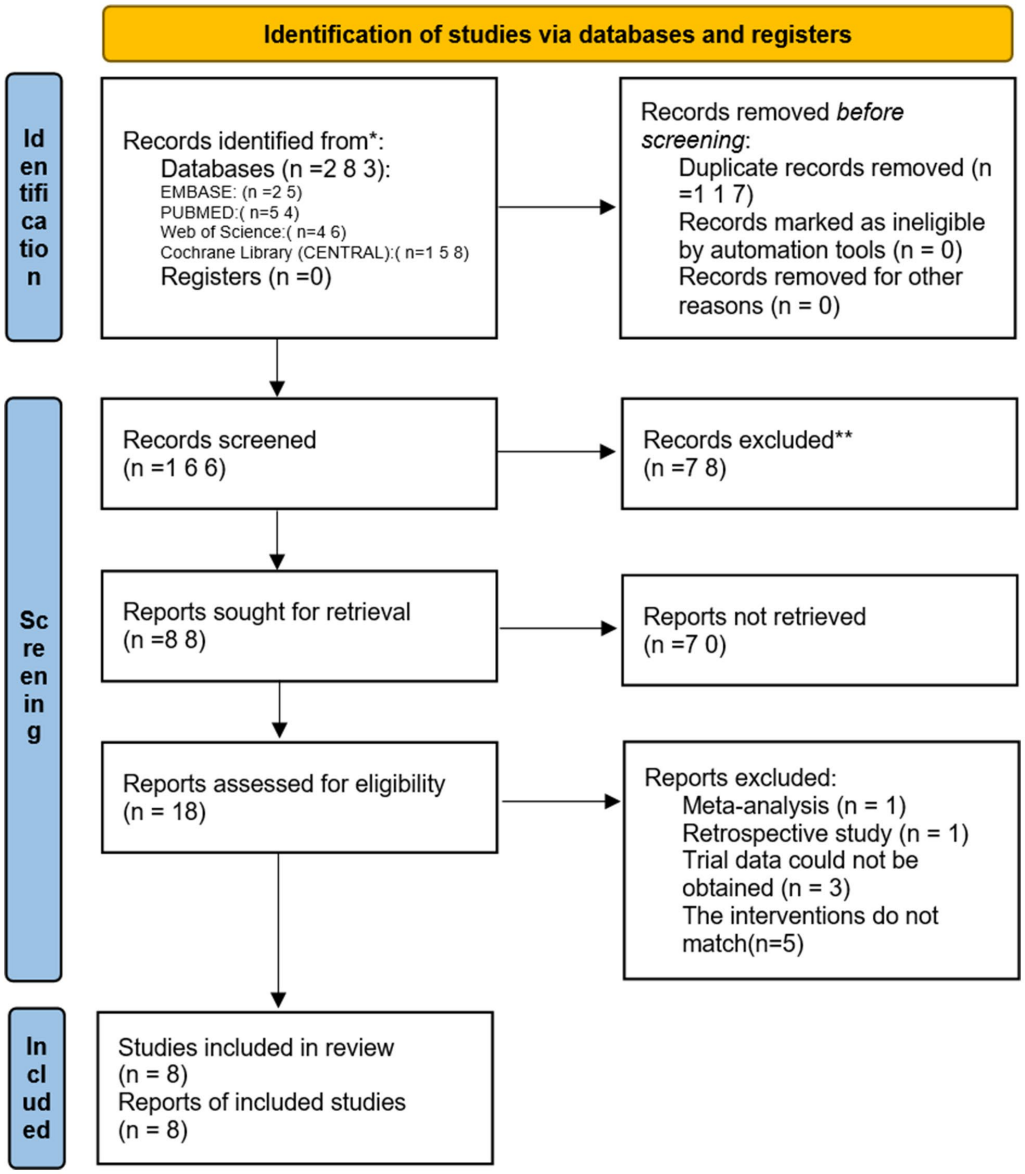
The PRISMA flow diagram.

Two reviewers independently searched PubMed, EMBASE, Cochrane Library, and Web of Science from the establishment of these databases until February 20, 2024. The search terms used were: “Remimazolam or ONO 2745 or ONO2745 or ONO-2745 or CNS 7056 or methyl 3-(8-bromo-1-methyl-6-(2-pyridinyl)-4H-imidazo (1,2-a)(1,4) benzodiazepin-4-yl) propanoate” AND “Aged or Elderly or Geriatric” AND “randomized controlled trial or randomized or placebo or RCT.” Additionally, trials that were unpublished or ongoing in the ClinicalTrials.gov database were searched, and other relevant studies were manually screened (Figure 1).

### 2.2 Inclusion and exclusion criteria

The inclusion criteria for this study are as follows: (1) elderly patients aged over 65 years who require general anesthesia with endotracheal intubation for surgical procedures; (2) the experimental group receives general anesthesia with remimazolam; (3) the control group receives general anesthesia with propofol. Other sedatives and analgesics may be used intraoperatively in both groups. (4) The primary or secondary outcomes must include the incidence of hypotension, MAP before and after induction, heart rate before and after induction, or the incidence of bradycardia; (5) the included studies must be randomized controlled trials (RCTs). Exclusion criteria: (1) studies for which data could not be extracted for analysis; (2) studies that have been published more than once; and (3) studies in which the control group is not treated with propofol.

### 2.3 Outcomes

Primary outcomes: incidence of hypotension, MAP before and after induction, incidence of bradycardia, and heart rate after induction.

Secondary outcomes: incidence of hypoxemia, nausea and vomiting, injection site pain, time to loss of consciousness (LOC), and extubation time.

### 2.4 Data extraction and assessment of risk of bias

Data extraction and quality assessment were independently conducted by two authors. In case of any disagreements, they consulted with the corresponding author, Linji Li. The following information was extracted: first author’s name, year of publication, country, average age of participants, sample size, type of surgery, surgery duration, interventions/comparisons, and other relevant outcome measures. The quality of the studies was assessed using the Cochrane Risk of Bias Tool. The level of certainty of the evidence was assessed using the Grading of Recommendations, Assessment, Development, and Evaluation (GRADE) system, and the final graphic was created using GRADEpro software (gradepro.org).

### 2.5 Statistical analysis

Data analysis was performed using Review Manager (version 5.3). Risk ratios (RR) and weighted mean differences (WMD) were used to analyze the data from binary and continuous groups, with 95% confidence intervals (CIs), and a *p*-value <0.05 considered statistically significant. Statistical heterogeneity was used to assess differences among the included studies. The *I*^2^ statistic was used to evaluate statistical heterogeneity: 0% ≤ *I*^2^ < 25% indicates no heterogeneity; 25% ≤ *I*^2^ < 50% indicates low heterogeneity; 50% ≤ *I*^2^ indicates moderate heterogeneity; 75% ≤ *I*^2^ ≤ 100% indicates high heterogeneity. If high heterogeneity (*I*^2^ ≥ 50%) was observed (11), sensitivity analysis was conducted using Stata version 16.0 to identify the sources of heterogeneity. Significant differences in study design, sample characteristics, or interventions may have contributed to high heterogeneity, and a random-effects model was chosen to better capture variability between studies. Additionally, when there was assumed variability in the true effect sizes between studies, suggesting that each study might estimate a different effect, a random-effects model was used. Otherwise, a fixed-effects model was applied. Publication bias was assessed using Egger’s test (12). We also performed meta-regression analysis to assess the impact of sample size and surgery duration on hemodynamics. These analyses were performed using CMA 3.0 software. Additionally, trial sequential analysis (TSA) was performed to estimate the required information size and evaluate the risks of type I and type II errors. A type I error of 0.05 and a type II error of 0.10 (power = 90%) were allowed. We performed the analysis using TSA 0.9 software.

## 3 Results

### 3.1 Identification and characteristics of the studies

**Figure 2:**
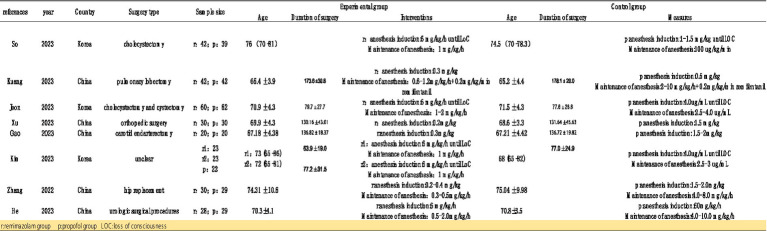
Features of included studies.

Initially, we identified 106 studies through database searches. Ultimately, 8 studies were included, with a total sample size of 571 participants: 298 in the remimazolam group and 273 in the propofol group (13–20). The studies were published between 2022 and 2023, and all were randomized controlled trials (RCTs) conducted in China and South Korea. Of these, 3 trials recorded the incidence of MAP, HR, and adverse events such as hypoxemia, nausea, vomiting, and injection pain before and after induction. Five studies reported the incidence of intraoperative hypotension and bradycardia. Four studies reported the duration of loss of consciousness (LOC) and extubation time (Figure 2).

### 3.2 Quality of the included studies

**Figure 3:**
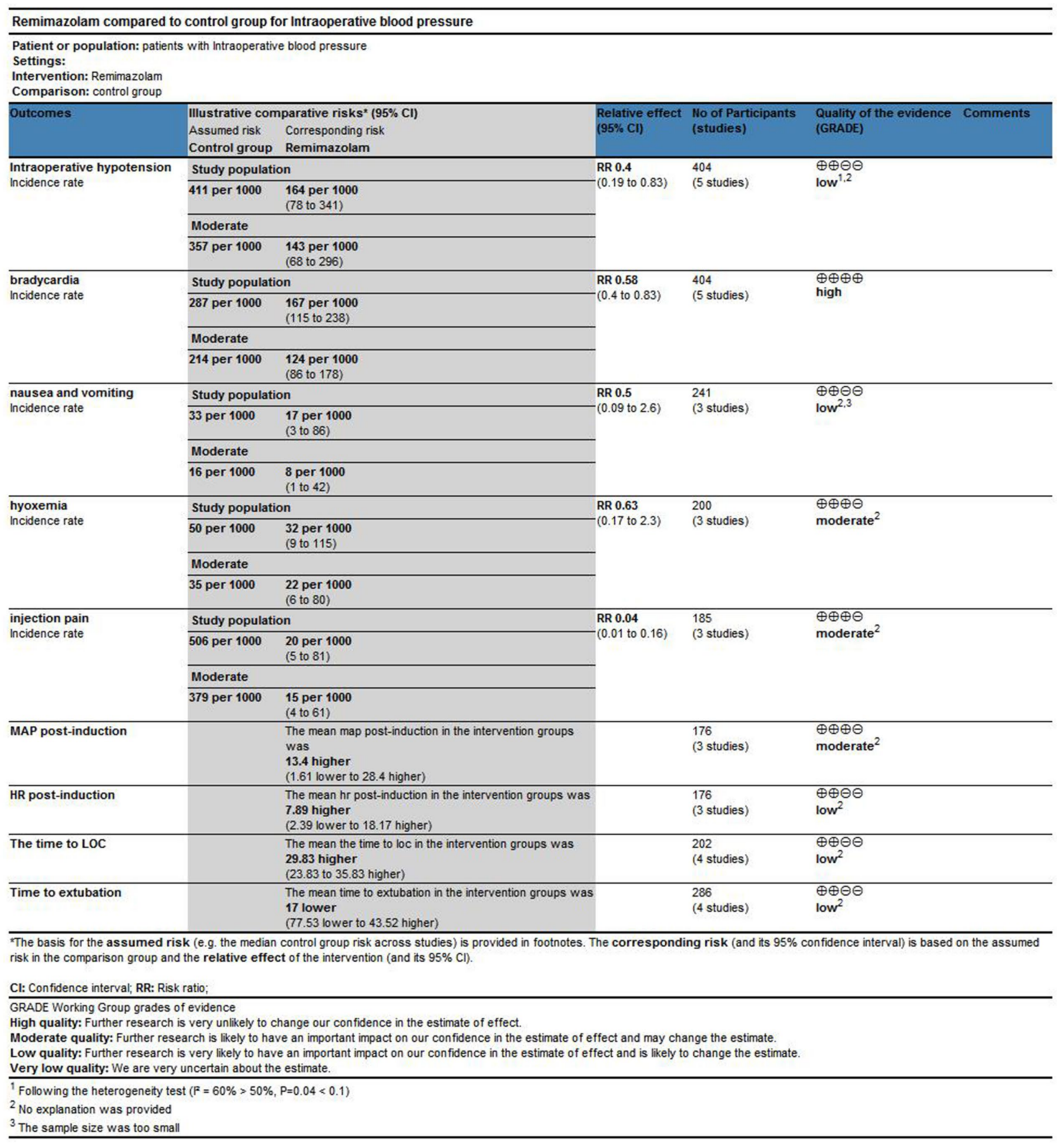
GRADE score table.

**Figure 4:**
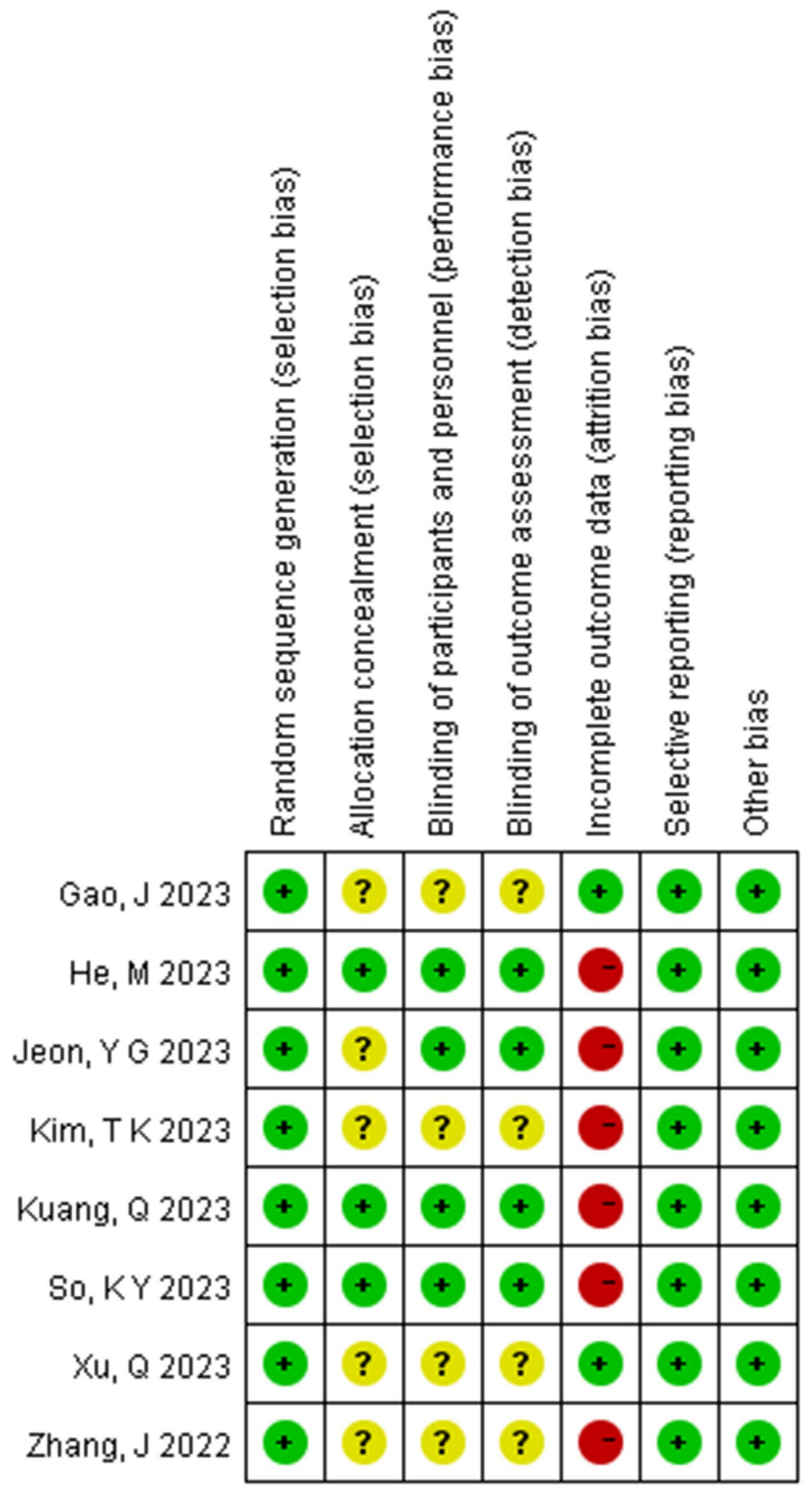
Summary of risk of bias across all trials (The figure includes seven bias risk assessments included in the study: green represents a low risk; yellow represents an unknown risk; red represents a high risk).

The risk of bias assessment results for the included studies are shown in Figure 4. Five studies were considered to have unclear risk regarding allocation concealment (15–19). Four studies were considered to have unclear risk regarding blinding (16–19). Six studies were considered to have a high risk regarding data integrity (13, 15, 16, 18–20), likely due to the poor general condition of elderly patients, which led to anesthesia cancelation or changes in the anesthesia plan. However, while the risk of bias assessment indicated that the overall quality of the included studies was reasonable, the GRADE evaluation showed that the quality of evidence for certain outcomes was low, such as the incidence of hypoxemia, nausea and vomiting, heart rate after induction, LOC time, and extubation time; this may be due to high heterogeneity and a limited number of studies (Figures 3, 4).

### 3.3 Primary outcome – hemodynamic stability

#### 3.3.1 Hypotension

**Figure 5:**
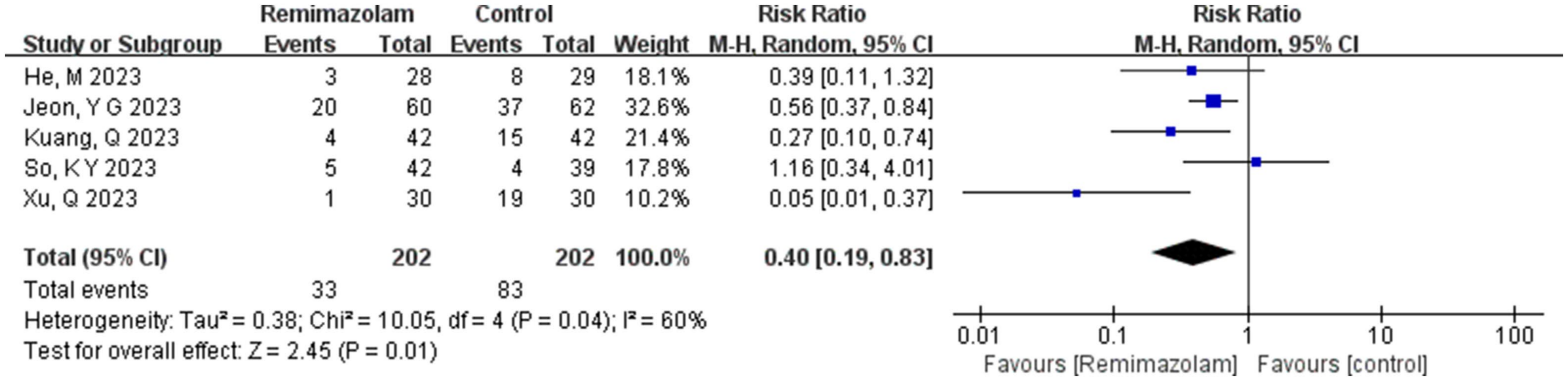
Summary of risk of bias across all trials.

**Figure 6:**
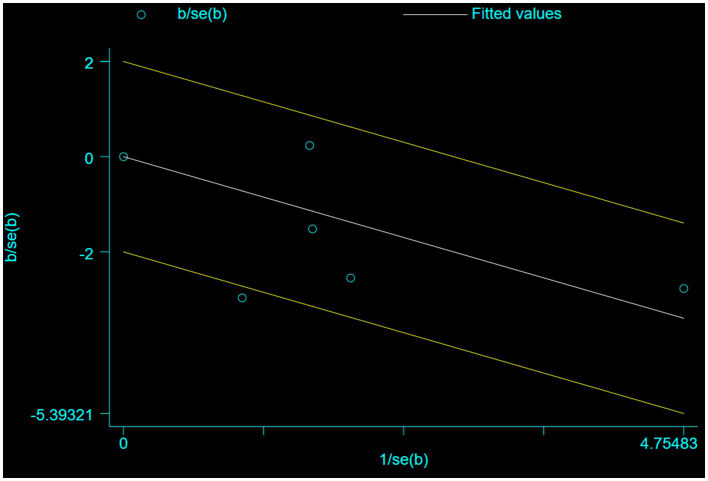
Galbraith plot of primary outcome - incidence of hypotension.

**Figure 7:**
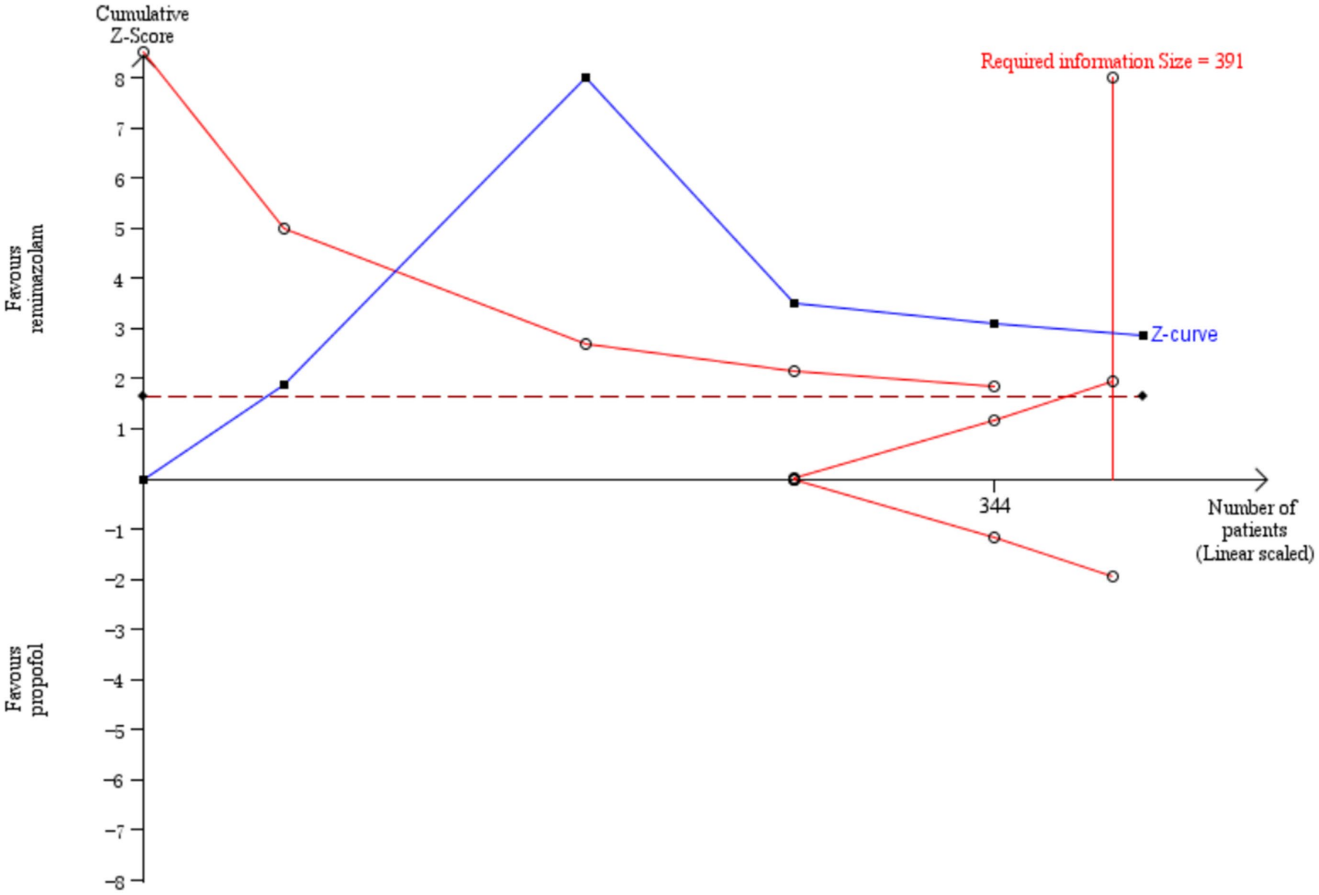
Incidence of hypotension by TSA.

**Figure 8:**
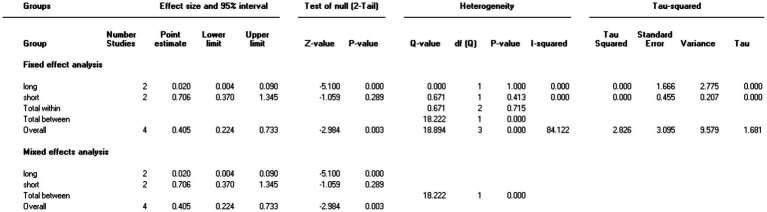
Meta-regression of the incidence of hypotension.

Five studies involving 404 elderly patients (13–16, 20) compared the incidence of hypotension between remimazolam (*N* = 202) and propofol (*N* = 202). After heterogeneity testing (*I*^2^ = 60%, *p* = 0.04), moderate heterogeneity was observed among the included studies. The random-effects model was used to pool the risk ratio (RR), resulting in (RR = 0.40, 95% CI: [0.19, 0.83], *Z* = 2.45, *p* = 0.01) as shown in Figure 5. This indicates that the incidence of hypotension in the remimazolam group was only 40% of that in the propofol group, and the difference was statistically significant. This suggests that remimazolam, compared to propofol, reduces the incidence of intraoperative hypotension in elderly patients under general anesthesia, and the result is statistically significant. The Egger’s test revealed no significant publication bias (*p* = 0.366). The Galbraith plot (Figure 6) showed a strong possibility of heterogeneity in one study. The study by Xu, Q, et al. exhibited significant heterogeneity compared to the others. After excluding this study, heterogeneity significantly decreased (*I*^2^ = 18%, *p* = 0.3), but the results remained unchanged (RR = 0.51, 95% CI: [0.33, 0.81], *Z* = 2.89, *p* = 0.04). The trial sequential analysis for hypotension incidence crossed the sequential monitoring boundaries and reached the required information size (power = 90%).We performed a meta-regression analysis on the incidence of hypotension and found that the duration of surgery significantly affected the incidence of intraoperative hypotension (Q = 18.222, *p* < 0.001) (Figures 7, 8).

#### 3.3.2 MAP post-induction

**Figure 9:**
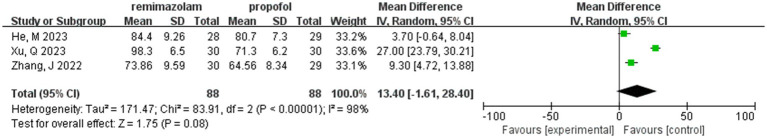
Forest MAP post-induction.

**Figure 10:**
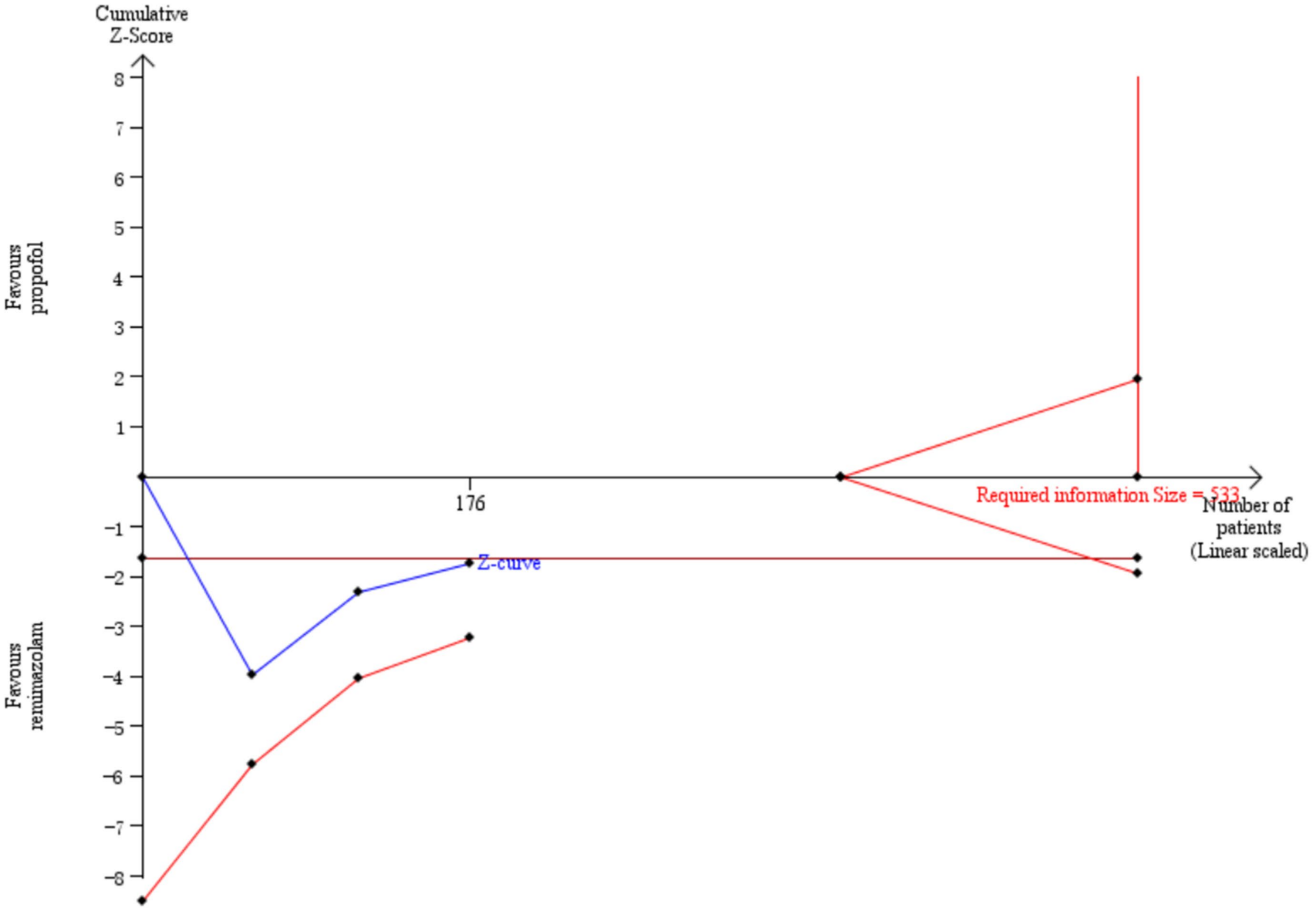
MAP post-induction by TSA.

Three studies involving 176 elderly patients (16, 19, 20) compared the differences in MAP before and after induction with remimazolam and propofol (remimazolam group, *N* = 88; propofol group, *N* = 88). Before conducting the meta-analysis, it was necessary to ensure baseline consistency between the two groups to proceed. The baseline consistency analysis showed no heterogeneity in the baseline MAP effect size (*I*^2^ = 0%, *p* = 0.701), indicating consistency between the two groups at baseline. The combined baseline effect size was-0.54 (*Z* = 0.39, *p* = 0.7), with no significant difference in baseline MAP between the two groups, allowing for further analysis. Three studies involving 176 elderly patients compared the changes in MAP before and after induction with remimazolam and propofol (remimazolam group, *N* = 88; propofol group, *N* = 88). However, there was high heterogeneity (*I*^2^ = 97.6%). The random-effects model yielded a combined effect size of 13.4, indicating that the remimazolam group had a significantly higher post-induction MAP than the propofol group, with a statistical significance (WMD = 1.75, 95% CI: [−1.609, 28.399], *Z* = 1.75, *p* = 0.08, < 0.05, see Figure 9). In the sensitivity analysis, after excluding the study by Xu, Q, two studies involving 116 elderly patients compared the MAP changes before and after induction with remimazolam and propofol (remimazolam group, *N* = 58; propofol group, *N* = 58). Heterogeneity significantly decreased (*I*^2^ = 67%), and although moderate heterogeneity remained, it was within an acceptable range. Using the random-effects model, the combined effect size was 6.35, indicating that the remimazolam group had a significantly higher post-induction MAP than the propofol group by 6.35 mmHg, with statistical significance (WMD = 3.95, 95% CI: [3.197, 9.498], *Z* = 3.95, *p* < 0.0001). The trial sequential analysis of post-induction MAP crossed the monitoring boundaries but did not reach the required information size (power = 90%). Therefore, the results may be falsely positive, and more trials are needed for validation (Figure 10).

#### 3.3.3 HR post-induction

**Figure 11:**
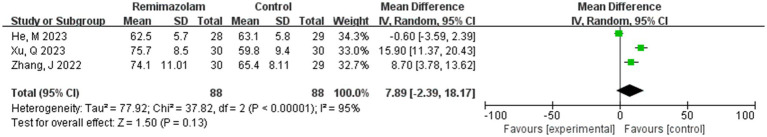
Forest plot of HR post-induction.

Three studies involving 176 elderly patients (16, 19, 20) compared the differences in heart rate (HR) after induction with remimazolam and propofol (remimazolam group, *N* = 88; propofol group, *N* = 88). Before conducting the meta-analysis, baseline consistency between the two groups must be ensured to proceed. The baseline consistency analysis showed no heterogeneity in the baseline HR effect size (*I*^2^ = 0%, *p* = 0.842), indicating no significant difference in baseline HR between the two groups, allowing for further analysis. Three studies involving 176 elderly patients compared HR differences after induction with remimazolam and propofol (remimazolam group, *N* = 88; propofol group, *N* = 88). After heterogeneity testing (*I*^2^ = 95%, *p* = 0.01), there was significant heterogeneity between the studies included in this analysis. A random-effects model was used, yielding a WMD of 7.89 (95% CI: [−2.39, 18.17], *Z* = 1.5, *p* = 0.13), indicating that the remimazolam group had a higher HR than the propofol group by 7.89, but the result was not statistically significant. We further performed a trial sequential analysis (TSA) on this result, but due to limited information, the analysis could not yield a conclusive result (Figure 11).

#### 3.3.4 Bradycardia

**Figure 12:**
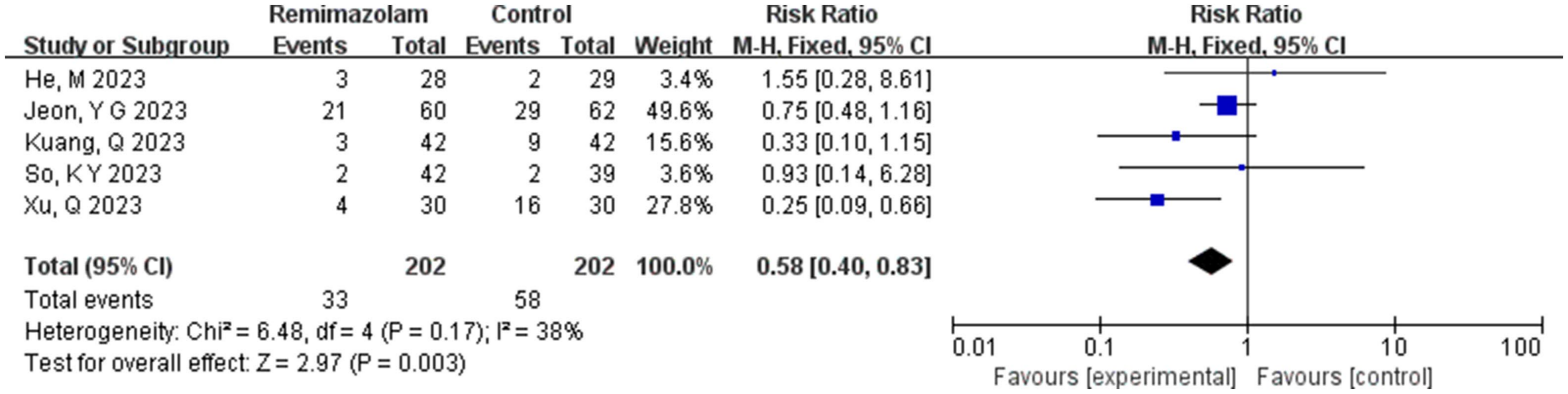
Forest plot of the incidence of bradycardia.

Five studies involving 404 elderly patients (13–16, 20) compared the incidence of hypotension between remimazolam and propofol (remimazolam group, *N* = 202; propofol group, *N* = 252). Heterogeneity testing showed low heterogeneity (*I*^2^ = 38%, *p* = 0.17), suggesting that the included studies were relatively homogeneous. A random-effects model was used, yielding a relative risk (RR) of 0.56 (95% CI: [0.31, 1.02], *Z* = 1.88, *p* = 0.06), meaning the incidence of hypotension in the remimazolam group was only 58% of that in the propofol group, which was statistically significant. This suggests that remimazolam may reduce the incidence of hypotension during general anesthesia in elderly patients compared to propofol. To explore the sources of heterogeneity, a sensitivity analysis was performed. We found that the study by Xu, Q, and colleagues contributed significant heterogeneity compared to the other studies. After removing this study, heterogeneity decreased significantly (*I*^2^ = 0%, *p* = 0.5), but the result was no longer statistically significant (RR = 0.72, 95% CI: [0.49, 1.07], *Z* = 1.62, *p* = 0.1). The trial sequential analysis (TSA) of the hypotension incidence crossed the monitoring boundaries but did not reach the required information size (power = 90%). Therefore, the result may be a false positive, and further trials are needed to validate the findings (Figure 12).

### 3.4 Secondary outcomes

#### 3.4.1 Nausea and vomiting

**Figure 13:**
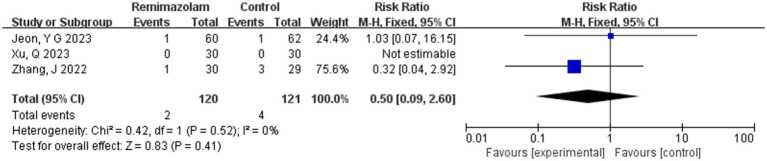
Forest plot of incidence of nausea and vomiting.

Three studies involving 241 elderly patients (15, 16, 19) compared the incidence of nausea and vomiting between remimazolam and propofol (remimazolam group, *N* = 120; propofol group, *N* = 121). Heterogeneity testing revealed no significant heterogeneity between the included studies (*I*^2^ = 0%, *p* = 0.52), so a fixed-effect model was applied. The relative risk (RR) was 0.50 (95% CI: [0.09, 2.60], *Z* = 0.83, *p* = 0.40), indicating that the incidence of nausea and vomiting in the remimazolam group was 50% of that in the propofol group. However, this result was not statistically significant (Figure 13).

#### 3.4.2 Hypoxemia

**Figure 14:**
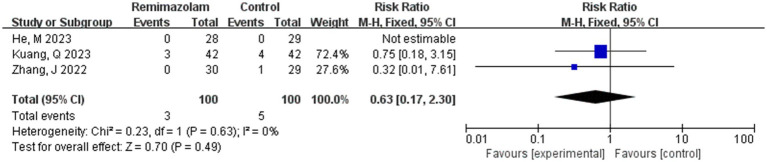
Forest plot of incidence of hypoxemia.

Three studies involving 200 elderly patients (14, 19, 20) compared the incidence of hypoxemia between the remimazolam group (*N* = 100) and the propofol group (*N* = 100). A heterogeneity test (*I*^2^ = 0% < 50%, *p* = 0.63 > 0.1) indicated no heterogeneity among the selected studies. Using a fixed-effects model, the results showed an RR of 0.63 (95% CI: [0.17, 2.30], *Z* = 0.70, *p* = 0.49 > 0.05, Figure 14), suggesting that the incidence of nausea and vomiting in the remimazolam group was 63% of that in the propofol group. However, this result was not statistically significant.

#### 3.4.3 LOC time

**Figure 15:**
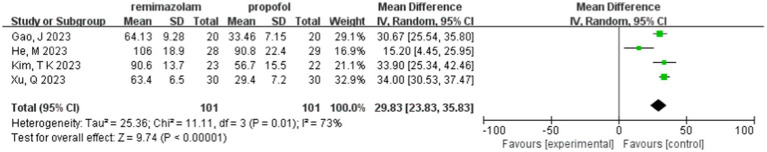
Forest plot of LOC time.

Four studies involving 202 elderly patients (16–18, 20) compared the time to loss of consciousness between the remimazolam group (*N* = 101) and the propofol group (*N* = 101). A heterogeneity test (*I*^2^ = 73%, *p* = 0.01 < 0.1) indicated significant heterogeneity among the selected studies. A random-effects model was used, yielding a WMD of 29.83 (95% CI: [22.83, 37.47], *Z* = 9.74, *p* < 0.00001). These results suggest that the time to loss of consciousness in the remimazolam group was 29.83 s longer than in the propofol group, a statistically significant difference. This implies that in elderly patients undergoing general anesthesia, remimazolam requires more time to induce loss of consciousness compared to propofol (Figure 15).

#### 3.4.4 Time to extubation

**Figure 16:**
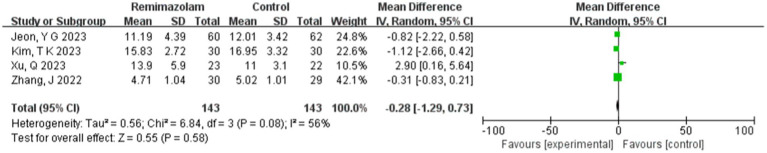
Forest plot of time to extubation.

Four studies involving 286 elderly patients (15, 16, 18, 19) compared the time to loss of consciousness (LOC) between the remimazolam group (*N* = 143) and the propofol group (*N* = 143). A heterogeneity test (*I*^2^ = 56%, *p* = 0.08 < 0.1) indicated the presence of heterogeneity among the included studies. A random-effects model analysis yielded a WMD of −0.28 (95% CI: [−1.29, 0.73], *Z* = 0.55, *p* = 0.58 > 0.05), suggesting that extubation time in the remimazolam group was 0.28 min shorter than in the propofol group. However, this result was not statistically significant. This indicates that remimazolam might shorten extubation time compared to propofol in elderly patients undergoing general anesthesia, but the difference lacks statistical significance. To explore the source of heterogeneity, sensitivity analysis was conducted, revealing that the study by Xu, Q. showed significant heterogeneity compared to the others. After excluding this study, heterogeneity decreased significantly (*I*^2^ = 0% < 50%, *p* = 0.52 > 0.1), but the result remained statistically insignificant (WMD = −0.26, 95% CI: [−0.54, 0.14], *Z* = 1.86, *p* = 0.52 > 0.05). Furthermore, Trial Sequential Analysis (TSA) was performed on LOC and extubation time, but due to limited information, no definitive conclusions could be drawn (Figure 16).

#### 3.4.5 Injection pain

**Figure 17:**
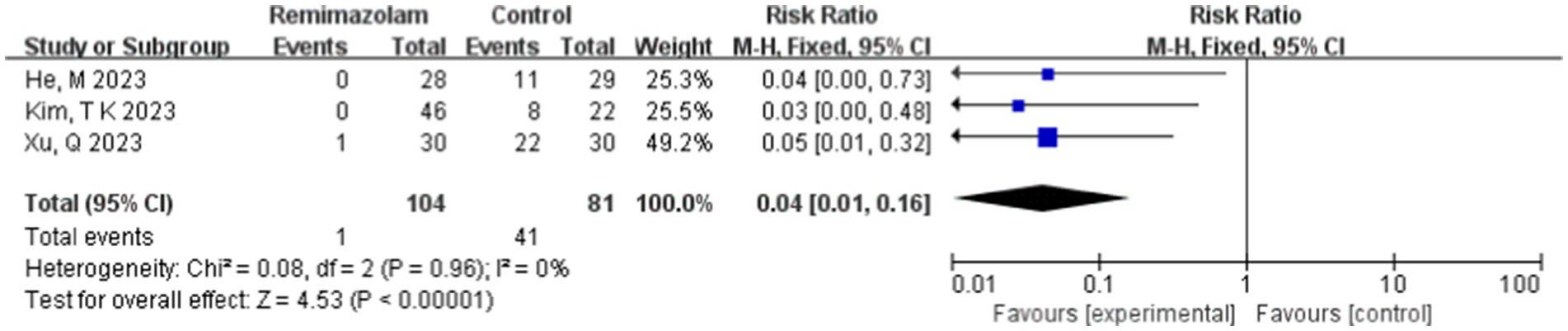
Forest plot of incidence of injection pain.

Three studies involving 185 elderly patients (16, 18, 20) compared the incidence of injection pain between the remimazolam group (*N* = 104) and the propofol group (*N* = 81). A heterogeneity test (*I*^2^ = 0% < 50%, *p* = 0.96 > 0.1) indicated no heterogeneity among the included studies. Using a fixed-effects model, the results showed an RR of 0.04 (95% CI: [0.01, 0.16], *Z* = 4.53, *p* < 0.00001, Figure 17). This suggests that the incidence of injection pain in the remimazolam group was only 4% of that in the propofol group, a statistically significant difference. These findings indicate that, for elderly patients undergoing general anesthesia, remimazolam significantly reduces the incidence of injection pain compared to propofol.

## 4 Discussion

The health conditions of elderly patients are often complicated by multiple comorbidities, making them less tolerant of anesthesia and surgery compared to younger patients. Maintaining hemodynamic stability during induction and improving blood perfusion to critical organs such as the heart, brain, and kidneys can enhance disease outcomes and prognosis in elderly patients (3, 21). Remimazolam, an ultra-short-acting intravenous benzodiazepine sedative, is characterized by rapid onset, quick recovery, and relatively minimal impact on hemodynamics. Its metabolism occurs via nonspecific esterases, meaning it does not impair liver or kidney function and does not accumulate with prolonged infusion. Additionally, its pharmacological effects are reversible and can be antagonized by flumazenil (22). Studies have shown that compared to propofol, remimazolam can be used safely and effectively for sedation during gastrointestinal endoscopy in elderly patients, with a lower incidence of sedation-related adverse events, particularly hemodynamic instability and respiratory depression (23–25). Its use in general anesthesia is also becoming increasingly widespread. Therefore, we conducted this meta-analysis to demonstrate that remimazolam may serve as a more effective sedative than propofol in general anesthesia for elderly patients.

In this meta-analysis, remimazolam significantly reduced the incidence of intraoperative hypotension (*p* = 0.01) and bradycardia (*p* = 0.03). However, some heterogeneity was observed in the results. Meta-regression analysis of hypotension revealed that surgery duration might significantly influence the incidence of intraoperative hypotension (*Q* = 18.222, *p* < 0.001), potentially explaining the source of heterogeneity. A meta-regression analysis of bradycardia incidence was also attempted, but no results were obtained due to an insufficient number of studies. Trial Sequential Analysis (TSA) for bradycardia incidence crossed the trial sequential monitoring boundary but did not meet the required information size (power = 90%), suggesting a potential false-positive result. Further trials are needed to confirm these findings. Propofol is commonly used for intravenous induction sedation in general anesthesia but is often associated with hemodynamic suppression. Previous studies have shown that propofol induces dose-dependent hypotension by reducing systemic vascular resistance and exerts negative inotropic effects on the myocardium. Elderly patients are particularly sensitive to the cardiovascular suppressive effects of propofol (26, 27). For elderly patients, most anesthetics exacerbate hypotension by causing systemic vasodilation. Due to impaired compensatory mechanisms, hypotension in the elderly can be particularly dangerous, potentially leading to myocardial injury, acute kidney injury (AKI), stroke, and death. The Perioperative Quality Initiative consensus statement on intraoperative hypotension recommends avoiding a mean arterial pressure (MAP) <65 mmHg and systolic blood pressure (SBP) <100 mmHg. The Anesthesia Society’s Best Practices for Perioperative Care in the Elderly further advises avoiding intraoperative hypotension in patients aged ≥65 years, defining it as a 20% reduction in SBP (28). Maintaining hemodynamic stability throughout surgery is crucial, underscoring the need for a safer and more effective sedative suitable for general anesthesia in elderly patients. In our study, we found that remimazolam was associated with a lower incidence of hypotension both during induction and intraoperatively. These results were robust in sensitivity analyses and TSA, aligning with previous findings and supporting the potential advantage of remimazolam as a more effective sedative.

Furthermore, this meta-analysis showed that the MAP (mean arterial pressure) significantly decreased in the propofol group compared to the remimazolam group after induction (*p* = 0.0001). However, the reliability of this result may be questionable due to significant heterogeneity among studies (*I*^2^ = 67%) and insufficient sample size. A meta-regression analysis was attempted to investigate this result, but no conclusive findings were obtained due to the limited number of included studies.

Our study found that the time to loss of consciousness (LOC) with remimazolam was longer, but there were significant differences across studies. In the studies by Gao J. and Xu Q., the LOC time for remimazolam was around 1 min, while for propofol it was approximately 30 s. In contrast, in the studies by Kim T. K. and He M., the LOC time for remimazolam was about 1 min and 30 s, while for propofol it was around 1 min. This discrepancy may be related to differences in drug dosages. Gao J. et al. used an induction dose of 0.3 mg/kg for remimazolam and 1.5–2 mg/kg for propofol; Xu Q. et al. used 0.2 mg/kg for remimazolam and 1.5 mg/kg for propofol; and in the studies by Kim T. K. and He M., the remimazolam induction dose was 0.1 mg/kg, while that of propofol was 1 mg/kg. Therefore, the high heterogeneity in LOC time may be attributable to differences in drug dosages among the included studies. Research suggests that the optimal induction dose for elderly patients aged 60–80 years is 0.19–0.25 mg/kg, while for those over 80 years, it is 0.14–0.19 mg/kg. Moreover, another meta-analysis found no significant difference in LOC time between remimazolam and propofol, which may be due to the use of additional anesthetic induction agents (29).

In recent years, several clinical studies have compared the incidence of adverse events such as hypoxemia, nausea, and vomiting in this context. However, the reporting of these outcomes has been inconsistent. For example, Doi, M. and colleagues compared the efficacy and safety of remimazolam and propofol for general anesthesia. Overall, the proportion of patients experiencing adverse drug reactions was higher in the propofol group (61.3%) than in the remimazolam group (41.0%). However, the frequency of nausea (7%) and vomiting (6%) was slightly higher in the remimazolam group compared to the propofol group (5.3 and 4.0%, respectively) (24). However, our meta-analysis did not find significant differences in the incidence of hypoxemia and nausea/vomiting between the two sedatives. We further performed a Trial Sequential Analysis (TSA) on these adverse events, but due to limited information, no conclusive results were obtained. This may be because the included studies involved heterogeneous patient populations, whereas our analysis focused exclusively on elderly patients. Another potential reason is the insufficient sample size in the included studies. We will continue to monitor research developments and update the meta-analysis results accordingly.

Due to the limited number of studies involving the use of flumazenil, the analysis of remimazolam reversal, as well as subgroup analysis and meta-regression, was restricted. In a recent meta-analysis involving adult patients undergoing general anesthesia with either propofol or remimazolam combined with flumazenil, Wu et al. found that the incidence of respiratory depression was lower in the remimazolam group compared to the propofol group. Considering that sedation reversal is a key characteristic of benzodiazepines, further investigation into the effects of flumazenil on remimazolam anesthesia is crucial (30).

Our meta-analysis also compared the incidence of injection site pain between remimazolam and propofol. Injection pain is one of the most commonly reported adverse effects associated with propofol, often causing significant discomfort and distress for patients. Several factors can explain the pain after propofol injection, including the irritant effect of phenol on the skin, mucosa, and venous endothelium. Additionally, delayed pain may result from the release of mediators such as kininogen as part of the kinin cascade (31). In contrast, remimazolam, a benzodiazepine with a completely different composition, is theoretically unlikely to cause injection pain. Studies have shown that intravenous administration of remimazolam can alleviate pain by blocking the bradykinin signaling pathway (32). Consequently, our findings, along with several recent studies, support the conclusion that the risk of injection pain is significantly lower with remimazolam compared to propofol (*p* < 0.00001).

Our meta-analysis suggests that remimazolam may be safer and more comfortable for elderly patients, with fewer adverse reactions. However, larger sample sizes are needed to confirm the reliability of these findings and provide more robust evidence for the benefits of remimazolam.

### 4.1 Limitations

This meta-analysis presents several limitations. Firstly, the sample size is relatively small, which could influence the outcomes of future analyses as more studies are reported. Secondly, the majority of the included studies originate from China and South Korea, potentially limiting the generalizability of the findings to broader patient populations. Thirdly, variations in surgical techniques, drug dosages, and other variables across the studies may contribute to substantial heterogeneity in certain outcome measures.

### 4.2 Conclusion

In conclusion, our study demonstrates that remimazolam is associated with a reduced frequency of adverse reactions during general anesthesia in elderly patients when compared to propofol, highlighting its potential clinical benefits. Additionally, the mean arterial pressure (MAP) remains more stable before and after induction with remimazolam, suggesting its enhanced suitability for sedation in this patient demographic.

## Data Availability

The original contributions presented in the study are included in the article/supplementary material, further inquiries can be directed to the corresponding author.
